# Resistin Activates p65 Pathway and Reduces Glycogen Content through Keratin 8

**DOI:** 10.1155/2020/9767926

**Published:** 2020-05-18

**Authors:** Fengyun Wen, Qiao Xia, Hui Zhang, Haipeng Shia, Amin Rajesh, Yanling Wu, Yi Yang, Zaiqing Yang

**Affiliations:** ^1^College of Animal Science and Technology, Henan University of Science and Technology, Luoyang 471000, Henan, China; ^2^Key Laboratory of Agricultural Animal Genetics, Breeding and Reproduction of Ministry of Education, College of Life Science and Technology, Huazhong Agricultural University, Wuhan 430070, Hubei, China; ^3^Department of Drug Discovery and Development, Harrison School of Pharmacy, Auburn University, 36849-5501 Auburn, Alabama, USA

## Abstract

Resistin is associated with metabolic syndrome and inflammatory conditions. Many studies have suggested that resistin inhibits the accumulation of glycogen; however, the exact mechanisms of resistin-induced decrease in glycogen content remain unclear. Keratin 8 is a typical epithelial intermediate filament protein, but numerous studies suggest a vital role of K8 in glucose metabolism. However, it is still not known whether K8 participates in the mediation of resistin-induced reduction of cellular glycogen accumulation. In this study, we found that resistin upregulated expression of the p65 subunit of NF-*κ*B, which led to the promotion of K8 transcriptional expression; in turn, the expression of K8 inhibited glycogen accumulation in HepG2 cells.

## 1. Introduction

Resistin is one of the important cytokines secreted by adipocytes. It was discovered in 2001 [1] and regarded as the link between obesity and type II diabetes mellitus (T2DM). Resistin is expressed at higher levels in obese humans and mice than in lean controls [2, 3]. Treatment with recombinant mouse resistin impaired the glucose tolerance and activity of insulin and induced hepatic insulin resistance [4]. Recombinant murine resistin significantly decreased glycogen content by decreasing the level of phosphorylated GSK-3*β* at Ser 9, leading to impaired hepatic insulin action [5]. Although many studies have attempted to clarify the mechanism of resistin-induced decrease in glycogen content, the relationship between resistin and glycogen remains unclear.

Keratin is a typical epithelial intermediate filament protein [6]. As a component of the epithelial cytoskeleton, keratin plays an important role in the maintenance of the stability and integrity of epithelial cells and tissues [7]. Some keratin proteins also serve as regulators in cell signaling pathways [8, 9]. Keratins are also used as biomarkers in the diagnosis of cancer [10]. Studies on keratin 8 have shown that it is an essential member of the keratin family, which plays an important role in glucose metabolism [9, 11]. Although problems with the uptake of glucose and its metabolites in hepatocytes are mainly attributed to chronic insulin-related disease, there is also evidence supporting a correlation between diabetes and various malignant tumors [12–15]. A similar correlation exists between glycogen storage disease and liver cell abnormality. The deletion of K8/K18 from hepatocytes promotes insulin-induced glycogen accumulation. Under high-glucose culture conditions, deletion or knockdown of K8/K18 in hepatocyte and hepatocellular carcinoma cells (HCCs) significantly promotes glycogen accumulation [9]. Other studies have found that K8 acetylation promotes perinuclear keratin filament organization. Acetylation also modulates K8 site-specific phosphorylation, a modification that is closely related to its solubility [16]. It is worth noting that the filament organization of K8 is regulated by glucose concentration [17]. In summary, the available data suggest a vital role of K8 in glucose metabolism. However, it is still not known whether K8 mediates resistin-induced reduction of cellular glycogen accumulation. In this study, we report that resistin upregulated p65, which inhibited the accumulation of glycogen in HepG2 cells by promoting the transcription of K8.

## 2. Materials and Methods

### 2.1. Materials

TRIzol reagent was purchased from Takara (Dalian, China), and Lipofectamine^TM^ 2000 was obtained from Invitrogen (Carlsbad, CA, USA). The antibody against the NF-*κ*B p65 subunit was purchased from Santa Cruz Biotechnology (Santa Cruz, CA, USA). Recombinant human resistin was purchased from PeproTech, Inc. (Rocky Hill, NJ, USA). The glucose assay kit was purchased from Applygen Technologies Co., Ltd. (Beijing, China). Small interfering RNAs (siRNAs) were synthesized by IBS Bio (Shanghai, China). The Dual-Luciferase Assay kit was purchased from Promega (Madison, WI, USA). The Chromatin Immunoprecipitation (ChIP) Assay kit was from Millipore (Burlington, MA, USA).

### 2.2. Animal Experiment

Male C57BL/6J mice (8 weeks old) were purchased from Huafukang Biotech (Beijing, China) and housed in individual plastic cages at around 25°C under a 12 : 12 h light-dark cycle with free access to water and food. Mice were divided into two groups (control and resistin-treated), 6 mice for each group. All of the mice were put on a standard chow and water diet. Animals in the group treated with resistin received 400 ng resistin daily for 6 days by injection via the vena caudalis [18]. After being fasted overnight, mice were sacrificed on day 7, and the livers were collected immediately and snap frozen in liquid nitrogen and then stored at −70°C before analysis. The Hubei Province Committee on Laboratory Animal Care approved all procedures.

### 2.3. Histology

Liver tissue samples (*n* = 6) were gathered 24 h after tail vein injection with or without resistin and fixed in 10% formalin solution. The samples were processed and embedded in paraffin. Hematoxylin and eosin (H&E) staining was used to identify and describe the different tissues and structures within them. Rehydrated slides were rinsed for 1 min in distilled water, immersed in hematoxylin (Servicebio, China) for 5 min, and rinsed in running tap water for 10 min. The slides were then rinsed in 80% ethanol for 1 min and immersed in eosin (Servicebio) for 5 min. Finally, the slides were dehydrated, cleared, and mounted with coverslips. Periodic acid-Schiff (PAS) staining was used to assess the glycogen content. Slides were rinsed in distilled water and immersed in PAS staining solution B (Servicebio) for 15 min, rinsed with distilled water, and immersed in PAS staining solution A (Servicebio) for 30 min. The slides were then rinsed in running tap water for 10 min, counterstained with PAS staining solution C for 30 s, rinsed, dehydrated, cleared, and mounted with coverslips.

### 2.4. Cell Culture

HepG2 (ATCC) cells were maintained in DMEM medium (HyClone, Thermo Scientific, Logan, UT, USA) supplemented with 10% fetal bovine serum (FBS) (Tianhang, Zhejiang, China), 100 U/mL penicillin, and 100 *μ*g/mL streptomycin and cultured at 37°C in a humidified chamber containing 5% CO_2_.

### 2.5. Vectors and Transfection

Expression vectors for p65 (pcDNA3.1-p65) were constructed by inserting the products of reverse transcription and PCR obtained from HepG2 RNA into pcDNA3.1. siRNAs for p65 and K8 were purchased from IBS (Shanghai, China). Unless otherwise indicated, all of the experiments were performed in three independent experiments, each in triplicate. Vectors and siRNAs were transfected into cells in a serum-free medium. The samples were prepared for transfection as follows: (A) to a 1.5 mL tube, we added 50 *µ*L opti-MEM medium, an appropriate amount of lipofectamine 2000, mixed gently, and left at room temperature for 5 min. (B) To another 1.5 mL tube, we added 50 *µ*L opti-MEM medium and diluted plasmid DNA, mixed gently, and left the mixture at room temperature for 5 min. (C) After 5 min, we gently mixed A and B and incubated the mixture at room temperature for 30 min to form the DNA-lipofectamine 2000 complex. Cells for transfection were washed three times with serum-free medium for 20 min, after which 100 *µ*L of the mixture containing the DNA-lipofectamine 2000 complex and 400 *µ*L opti-MEM were added. The cells were then incubated at 37°C for 6 h, and then the medium exchanged for growth medium. The cells were harvested for 24 h after transfection to isolate RNA or protein.

### 2.6. RNA Isolation

Total RNA was extracted with Trizol reagent (Takara, Dalian, China). Briefly, 500 *µ*L Trizol reagent was added to each well in a 24-well plate. Cells were collected and 200 *μ*L of chloroform was added to it and centrifuged at 12000 rpm for 15 min. Then, the upper phases were collected, and equal volumes of isopropanol were added and centrifuged at 12000 rpm for 10 min. The precipitate was washed twice with 500 *µ*L of 75% ethanol, centrifuged at 8000 rpm for 5 min, air-dried, dissolved with 30−50 *µ*L of DEPC water, and stored at −80°C. The RNA quality was assessed using a UV-Vis spectrophotometer (SMA4000). RNA was generally of high quality (an average 260/280 ratio of 2.02 and an average 260/230 ratio of 1.79).

### 2.7. cDNA Reverse Transcription

The following reaction mixture (30 *µ*L) was prepared in 1.5 mL tubes: RNA sample, 5 *µ*L; oligo T18 (20 nmol/L), 3 *µ*L; dNTP (20 nmol/L), 3 *µ*L; M-MLV reverse transcriptase, 0.5 *µ*L; RNase inhibitor, 0.5 *µ*L; 5× M-MLV buffer, 6 *µ*L; and DEPC water, 12 *µ*L. We incubated the mixture for 1 h at 42°C and 90°C for 10 min to stop the reaction and stored the sample at −20°C.

### 2.8. Quantitative Real-Time PCR

For real-time PCR, we followed the method of Wen et al. [19]. The sequences of the primers used for qRT-PCR are listed in Table 1.

### 2.9. Luciferase Assays

Cells were seeded into 24-well plates at a concentration of 1.0∼2.5 × 10^3^ cells and allowed to grow overnight. When it comes up to 80% confluency, the cells were incubated in serum-free DMEM for 6 h before transfection. We cotransfected the recombinant vector (pGL3-K8 promoter) or empty vector (pGL3-basic) with p65-expression plasmids (pcDNA3.1-p65) into the cells using Lipofectamine™ 2000 (Invitrogen) following the manufacturer's protocol. The pGL3-basic vector, containing the Firefly luciferase reporter, was used for normalization. After 24 h, Firefly and Renilla luciferase activities were measured consecutively using the dual-luciferase assay. Three independent experiments were performed each in triplicate.

### 2.10. Measurement of Glucose and Glycogen Content

The glucose concentration in the medium was assayed using the Glucose Assay kit (Applygen). Absorbance was measured at 500 nm using a Beckman Coulter DU 800 UV-visible spectrophotometer. All of the samples were normalized to the total amount of protein. The glycogen level in each group was assessed using a glycogen assay kit (Nanjing Jiancheng Bioengineering Institute, China), and the results were normalized to a standard curve and expressed as milligrams of glycogen per milliliter of protein, as per the manufacturer's instructions.

### 2.11. Chromatin Immunoprecipitation

Chromatin immunoprecipitation (ChIP) assays were performed using Chromatin Immunoprecipitation Kits (Millipore) according to the manufacturer's instructions. Briefly, HepG2 cells were seeded 24 h before and fixed with 1% formaldehyde for 15−20 min to crosslink the chromatin; the reaction was then stopped by adding glycine to a final concentration of 0.125 M. The cells were disaggregated by homogenizing, passed through a 200 *μ*m pore filter, and centrifuged at 1000 rpm for 5 min at 4°C. The cell pellets were resuspended in 500 *µ*L cell lysis buffer and 2.5 *µ*L protease inhibitor, incubated on ice for 15 min, and centrifuged at 800*g* for 5 min at 4°C. The supernatants were discarded, and the cells were resuspended in 500 *µ*L nuclear lysis buffer and 2.5 *µ*L protease inhibitor. The cells were then sonicated to break the DNA into 200–1000 bp fragments, followed by 10,000*g* centrifugation for 10 min at 4°C to remove insoluble substances. For each IP reaction, we prepared a mixture in a 1.5 mL tube as follows: dilution buffer, 450 *µ*L; protease inhibitor, 2.25 *µ*L; DNA fragments, 50 *µ*L; primary antibody, 2 *µ*g; and protein G magnetic beads, 20 *µ*L. We incubated the samples at 4°C overnight with constant rotation and collected the protein G-antibody complex magnetically and removed the supernatants the next day. We washed the protein G with 500 *µ*L of low-salt immune complex wash buffer, high-salt immune complex wash buffer, LiCl immune complex wash buffer, and TE buffer, respectively. We added 100 *µ*L of ChIP elution buffer and 1 *µ*L of protease K and incubated at 62°C for 2 h and then at 95°C for 10 min. We transferred the protein G complex to a new tube, added 500 *µ*L of binding reagent A, applied the sample on a column with a collection tube, and centrifuged at 10,000*g* for 30 s. We then washed the column with 500 *µ*L of wash buffer B and eluted with 50 *µ*L of elution buffer C. We purified the DNA for use as the template for PCR. The PCR was carried out under the following conditions: 95°C for 5 min followed by 30 cycles of 20 s at 94°C, 30 s at 60°C, and 30 s at 72°C and a 5 min final extension at 72°C. The primers used for ChIP assay are listed in Table 2.

### 2.12. Statistical Analysis

Data are presented as means ± standard deviation (SD). Statistical analyses were performed by GraphPad Prism 5.0 using the unpaired two-tailed *t*-test (for two groups) and analysis of variance (ANOVA; for multiple groups). *P* values <0.05 were considered to be statistically significant.

## 3. Results

### 3.1. Resistin Downregulates Glycogen Levels Both in Liver and HepG2 Cells

In our previous study, the levels of blood glucose, insulin, and triglycerides were found to significantly increase in C57BL/6J mice treated with resistin for six days [19]. In this study, we aimed to investigate the effects of resistin on glycogen storage.

**Figure 1:**
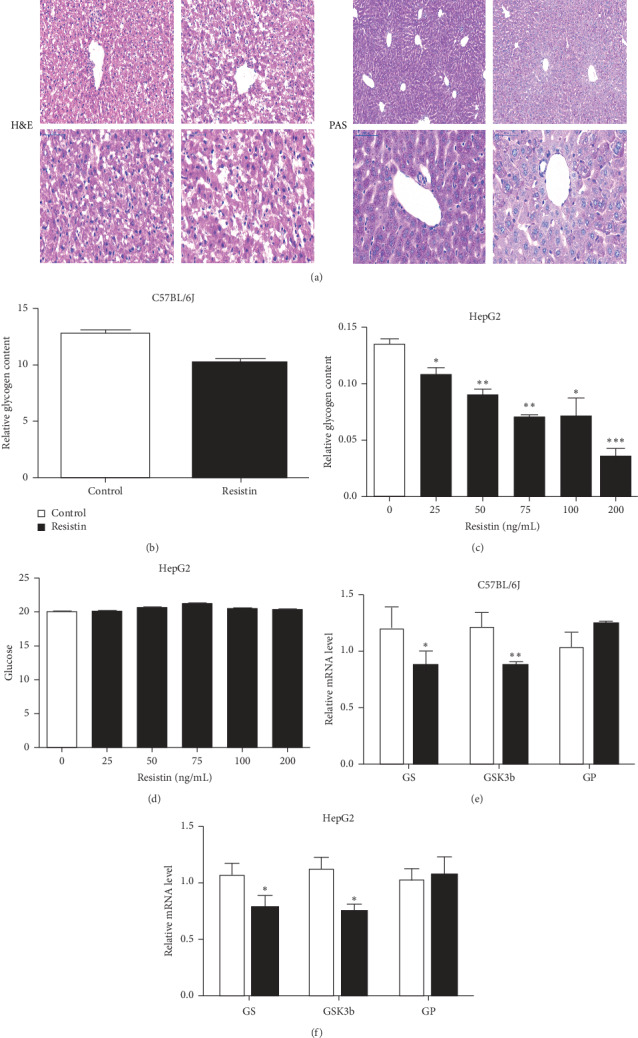
Resistin inhibits the production of glycogen. Eight-week-old male C57BL/6J mice were treated with or without resistin, and the PAS staining (a) and the glycogen content (b) measured by the glycogen assay kit (Nanjing Jiancheng Bioengineering Institute, China) were used to assess the glycogen content directly. HepG2 cells were treated with human recombinant resistin protein, and the glycogen content in cells (c) and glucose levels in the medium (d) measured. Eight-week-old male C57BL/6J mice (e) or HepG2 cells (f) were treated with or without resistin and the genes related to glycogen metabolism (GS, GSK3*β*, and GP) were analyzed by using qRT-PCR. Data are presented as mean ± SD. ^*∗*^*p* < 0.05 and ^*∗∗*^*p* < 0.01.

Eight-week-old male C57BL/6J mice were treated with or without resistin, and glycogen in the liver was measured using the kit (Nanjing Jiancheng Bioengineering Institute, China). Liver tissue slices were stained with H&E, which showed that there were more numerous and larger vacuoles in the hepatic cytoplasm of the resistin-treated group than in the control group (Figure 1(a)). PAS staining was used to assess the glycogen content directly. Both PAS staining and measurement of the glycogen content showed that the glycogen content was significantly decreased in the livers of resistin-treated mice compared with controls (Figures 1(a) and 1(b)).

To verify the result in vitro, we treated HepG2 cells with human recombinant resistin protein. The results showed that treatment of HepG2 cells with different concentrations of resistin for 24 h resulted in substantial inhibition of cellular glycogen accumulation. Although the accumulation amount of glycogen was inversely correlated with resistin dosage (Figure 1(c)), even a low concentration (25 ng/mL) of resistin resulted in a significant reduction of glycogen levels.

Previous studies reported that resistin suppresses insulin-mediated cellular glucose uptake [20, 21]. To clarify whether the reduction in cellular glycogen content was due to resistin-mediated inhibition of cellular glucose uptake, we measured the glucose content in HepG2 cell medium 12 h after resistin treatment. The results showed that resistin did not significantly affect glucose uptake in the absence of insulin (Figure 1(d)), yet considerably lowered the glycogen content. These findings suggest that the action of resistin on glycogen accumulation is mediated through a mechanism other than lowering glucose uptake.

Furthermore, the expression of genes related to glycogen metabolism was examined in both mice and HepG2 cells treated with or without resistin using qRT-PCR. The results showed that glycogen synthase (GS) and glycogen kinase 3 beta (GSK-3*β*) were significantly decreased in the treated mice liver and HepG2 cells, but the glycogen phosphorylase (GP) was not affected by resistin administration (Figures 1(e) and 1(f)).

### 3.2. Role of K8 in Resistin-Regulated Glycogen Biosynthesis

**Figure 2:**
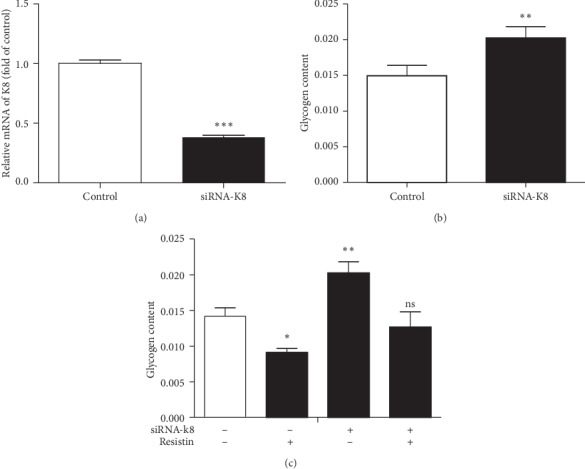
Resistin decreases glycogen biogenesis by regulating K8. HepG2 cells were transfected with K8 siRNA. The levels of keratin messenger (a) and the glycogen content (b) were determined. The cellular glycogen content was measured with or without resistin for 24 h after transfection with K8 (c). Data are presented as mean ± SD. ^*∗*^*p* < 0.05 and ^*∗∗*^*p* < 0.01.

Since previous data have suggested a vital role for K8 in glucose metabolism, we examined the interaction between resistin and K8 in regulating glycogen biogenesis. HepG2 cells were transfected with K8 siRNAs (Figure 2(a)), which resulted in a significant increase in glycogen accumulation after 24 h (Figure 2(b)). We then treated HepG2 cells with resistin for 24 h after K8 knockdown and measured the glycogen accumulation in the cells. Resistin significantly counteracted the increased glycogen accumulation caused by K8 ablation (Figure 2(c)). In summary, these findings indicate that inhibition of glycogen accumulation caused by resistin is partly due to the action of K8.

### 3.3. p65 Directly Binds to the K8 Promoter

**Figure 3:**
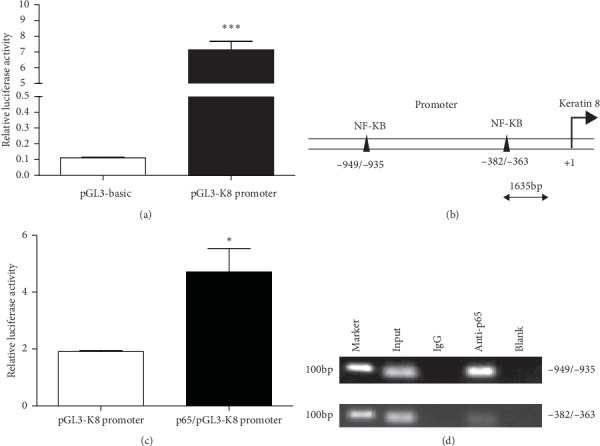
p65 binds directly to the K8 promoter. (a) The luciferase activity of the plasmid containing the pGL3-K8 promoter was greatly increased compared with that of control. (b) Schematic of the putative K8 promoter with two potential p65 response elements. (c) Luciferase assays in HEK293A cells. p65 significantly increased the luciferase activity of the vector containing the pGL3-K8 promoter. (d) Chromatin immunoprecipitation assays revealed that p65 interacts with the K8 promoter. Data are presented as mean ± SD. ^*∗*^*p* < 0.05 and ^*∗∗*^*p* < 0.01.

To determine the mechanism whereby resistin regulates K8, we constructed a dual-luciferase reporter vector containing the K8 promoter (Figure 3(a)). We analyzed the 1635 bp K8 promoter sequence using software from Genomatix (http://www.genomatix.de/) and identified potential transcription factor binding sites. A total of five NF*κ*B binding sites were found on the K8 promoter, two of which partially overlapped and were subject to analysis as one site (Figure 3(b)). The p65 protein, a subunit of NF-*κ*B, is a nuclear transcription factor with roles in the regulation of many cellular processes. However, it is still not known whether it directly regulates K8 transcription. The dual-luciferase reporter vector containing the K8 promoter (pGL3-K8 promoter) or the empty vector (pGL3-basic) was transfected into the cells together with p65-expressing plasmids (pcDNA3.1-p65). A luciferase assay showed that p65 significantly enhanced K8 promoter activity (Figure 3(c)).

To further validate the direct binding of p65 to the K8 promoter, we conducted a ChIP assay. The results showed that p65 bound directly to the −949/−935 and −382/−363 sites on the K8 promoter region and regulated its transcription (Figure 3(d)).

### 3.4. Resistin Regulates K8 Transcription through p65

**Figure 4:**
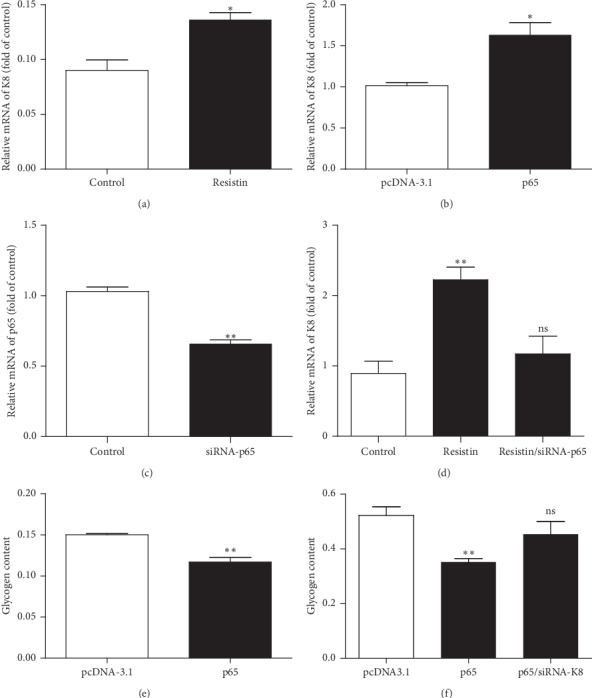
Resistin regulates K8 transcription through p65. (a) HepG2 cells were treated with or without resistin and K8 expression detected by using qRT-PCR. (b) HepG2 cells were transfected with a plasmid overexpressing p65 and K8 expression assessed by using qRT-PCR. (c) HepG2 cells were transfected with siRNA-p65 and p65 expression was determined by using qRT-PCR. (d) HepG2 cells were treated with or without resistin for 24 h after transfection with siRNA-p65, and K8 mRNA levels were determined by using qRT-PCR. (e) HepG2 cells were transfected with p65, and the glycogen content was determined after 24 h. (f) HepG2 cells were cotransfected with K8 siRNA and the p65 expression plasmid, and the cellular glycogen content was measured after 24 h. Data are presented as mean ± SD. ^*∗*^*p* < 0.05 and ^*∗∗*^*p* < 0.01.

To determine whether p65 is involved in resistin-mediated transcriptional regulation of K8, we measured K8 expression in HepG2 cells treated with resistin and transfected with an expression plasmid for p65. Both p65 overexpression and resistin treatment were found to induce significant upregulation of K8 mRNA (Figures 4(a) and 4(b)). To verify that resistin regulates the transcription of K8 through p65, we transfected HepG2 cells with p65 siRNA (Figure 4(c)) and then treated the cells with resistin for 24 h. Knockdown of p65 suppressed resistin-induced upregulation of K8 mRNA (Figure 4(d)), indicating that resistin regulated K8 transcription activity through p65.

### 3.5. P65 Is Involved in the Glycogen Accumulation Regulated by Resistin through K8

**Figure 5:**
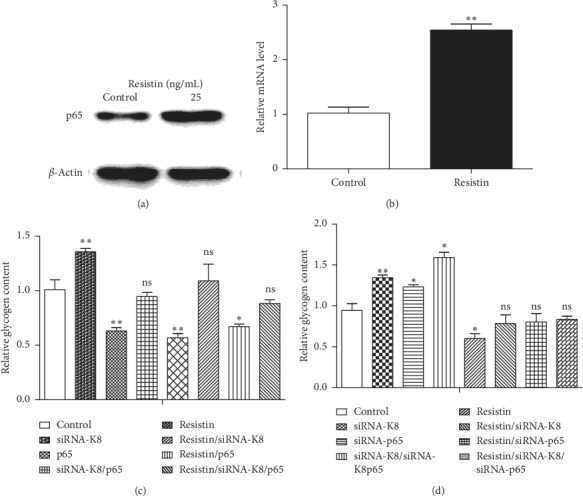
Resistin-regulated accumulation of glycogen through K8 involves p65. HepG2 cells were treated with human recombinant resistin protein, and the protein level (a) and mRNA level (b) of p65 were determined by using Western blot and qRT-PCR, respectively. (c) HepG2 cells were treated with or without resistin after cotransfection with K8 siRNA and p65 expression plasmid, and the glycogen content determined. (d) HepG2 cells were treated with or without resistin after cotransfection with K8 siRNA and p65 siRNA, and the glycogen content was determined. Data are presented as mean ± SD. ^*∗*^*p* < 0.05 and ^*∗∗*^*p* < 0.01.

To clarify whether the downregulation of glycogen content by resistin is mediated through the binding of p65 to the promoter of K8, we first examined the effect of resistin on the levels of p65 mRNA and protein expression by using qRT-PCR and Western blot, respectively. The results were consistent with previous findings showing that resistin stimulated the p65 expression both at the mRNA level and protein level (Figures 5(a) and 5(b)). We then transfected HepG2 cells with pcDNA3.1-p65/siRNA-p65 and siRNA-K8 alone or in combination, with or without treatment with resistin, and measured the glycogen content. The results showed that resistin regulated the glycogen content in a K8-dependent manner (Figures 5(c) and 5(d)).

## 4. Discussion

As an important component of the cytoskeleton family, keratin has been widely studied since the 1970s [21–23]. However, early studies mainly focused on the structure and biological diversity of keratin instead of functional analysis. Although keratins are extensively used as biomarkers in the diagnosis of cancer [24], studies on their functions other than their role in maintaining structure are still rare. In recent years, keratins have been found to play key roles in cell apoptosis [24, 25], cell growth [26], and wound healing [27]. Deletion of K8/K18 in normal and cancerous mouse hepatic cells promotes insulin-mediated glucose uptake [28], suggesting that keratin plays a role in metabolism. The regulatory role of keratin in resistin-mediated glycogen accumulation is the basis of this study.

This study investigated the relationship between resistin and keratin 8 in the regulation of glucose metabolism. Glycogen accumulation in hepatic cells was inhibited by a wide range of resistin concentrations (from 25 ng/mL, which is the concentration in normal individuals, to 200 ng/mL, the concentration in diabetic patients), with significant inhibition occurring in response to lower resistin concentrations. Thus, in this study, we used a low level (25 ng/mL) of resistin to stay within the physiologically normal range. Several groups have reported a regulatory role of K8/18 in glucose metabolism; however, the specific mechanism has not yet been addressed. In this study, we found that resistin regulated glycogen accumulation in hepatic cells through K8, providing additional evidence of the role of keratin in glucose metabolism.

Many studies have demonstrated a relationship between resistin and inflammation. Human resistin stimulates the proinflammatory cytokines TNF-*α* and IL-12 in macrophages through an NF-*κ*B-dependent pathway [29], while exogenous resistin promotes the dimerization of p50/p65 and its nuclear translocation. Resistin can also upregulate p65 mRNA expression and protein level [30, 31]. We also confirmed that human resistin increased p65 expression in a dose-dependent manner [32] and that p65 overexpression substantially inhibited glycogen accumulation in HepG2 cells. However, the silencing of K8 significantly reversed the inhibition of glycogen accumulation induced by p65 overexpression, indicating that p65 could regulate cellular glycogen accumulation by directly controlling K8 transcriptional expression. Our findings confirm the relationship between resistin and inflammation through keratin and shed new light on the mechanism of resistin-mediated glycogen accumulation. Interestingly, although both resistin and p65 overexpression can decrease glycogen, we did not find superimposed effects with resistin and p65 in inhibiting glycogen content (Figure 5(c)). One possible explanation is that a supplementary mechanism exists, which maintains cellular homeostasis; such a mechanism remains to be verified.

Previous reports have suggested that K8 acetylation promotes perinuclear keratin filament organization [17]. Acetylation also modulates K8 site-specific phosphorylation, which regulates K8 solubility [17]. In vitro, glucose promotes K8 acetylation, while K8 acetylation itself has been found to be increased in diabetic mice [28]. Resistin-induced inhibition of cellular glycogen accumulation is an outcome of multiple regulatory processes that vary under different physiological conditions. However, it is still not known if resistin affects K8 filament reorganization at the protein level or if filament reorganization plays a dominant role in the regulation of glycogen accumulation. The answers to these questions will help explain why resistin has multiple functions in glucose metabolism and facilitate the development of novel antidiabetic drugs.

In summary, we found that resistin upregulated p65 expression, while p65 promoted K8 transcriptional expression by directly binding to the K8 promoter, which inhibited cellular glycogen accumulation.

## Figures and Tables

**Table 1:** The primers for real-time PCR amplifications.

Gene name	Sequence	Size (bp)	Accession number
Homo GAPDH	F: TGCACCACCAACTGCTTAGC	147	NM_002046
	R: GGCATGGACTGTGGTCATGAG		

Homo p65	F: ATCCCATCTTTGACAATCGTGC	146	NM_021975
	R: CGTGAAATACACCTCAATGTCCTC		

Homo Keratin 8	F: GCCGTGGTTGTGAAGAA	165	NM_001256282.1
	R: CTGTTCCCAGTGCTACCCT		

Homo GP	F: TGATGGCAGCCACTCTACGA	97	AH002957
	R: TCACAGTCCGAGGCACAAAA		

Homo GS	F: ACAAGCAGTGCGAAAACAGC	199	S70004
	R: CATGTTGTGCGTGGTCACTG		

Homo GSK3*β*	F: GCAGCAAGGTGACAACAGTG	149	NM_002093
	R: GGCGACCAGTTCTCCTGAAT		

Mus GP	F: CCTTCGCCTACACCAACCAC	162	NM_133198.2
	R: TGCGGCTGATGTCTTTAGGA		

Mus GS	F: AGGACATTTCAGGGATTAA	176	NM_030678.3
	R: CGTCTACCTCTACCTACCG		

Mus GSK3*β*	F: ACCCTCATTACCTGACCTT	154	NM_019827.7
	R: TCGGCAGACAATTCAACTC		

**Table 2:** Primers for ChIP assay.

Name	Sequence (5′ ⟶ 3′)	Size (bp)
ChIP-p65-363	F: CCCTCAGTCTTTGTTATTCCTCG	85
	R: CAGCACAGTATTCTCCTTTCCTA	

ChIP-p65-935	F: CCTACAGCATATCTGTTACGT	89
	R: GGCAAGCTACTAGAACTCACT	

## Data Availability

The data used to support the findings of this study are available from the corresponding author upon request.
